# Multidrug-resistant *Mycolicibacterium fortuitum* infection in a companion cat (*Felis silvestris catus*) in Brazil

**DOI:** 10.1099/acmi.0.000317

**Published:** 2022-02-28

**Authors:** Sergio Morgado, Nilcéia de Veiga Ramos, Bárbara Bianca do Nascimento Pereira, Fernanda Freitas, Érica Lourenço da Fonseca, Ana Carolina Vicente

**Affiliations:** ^1^​ Fundação Oswaldo Cruz, Instituto Oswaldo Cruz, Laboratório de Genética Molecular de Microrganismos, Rio de Janeiro, Brazil; ^2^​ Universidade de Vassouras, Faculdade de Ciências Médicas de Maricá, Rio de Janeiro, Brazil; ^3^​ Clinicat, Rio de Janeiro, Brazil

**Keywords:** Zoonosis, micobacteria, genome, pathogen, animal, veterinary

## Abstract

*
Mycolicibacterium fortuitum* is a fast-growing bacterium and an opportunistic pathogen implicated in human and animal infections. Here we report the first case and genetic characterization of a strain of *M. fortuitum* isolated from skin lesions of a companion cat with atypical cutaneous mycobacteriosis in Brazil. In addition, the genome of this strain was sequenced, representing the first genome of this opportunistic pathogen isolated from an animal infection. The *in silico* and *in vitro* analysis regarding antibiotic resistance of this strain showed an intrinsic multiresistance antibiotic profile. However, this strain showed sensitivity to amikacin and ciprofloxacin, and the cat was treated long-term with these drugs.

## Introduction

*Mycolicibacterium fortuitum* (also known as *Mycobacterium fortuitum *) is a fast-growing non-tuberculous mycobacteria (NTM) that is saprophytic and ubiquitous in the environment (found in soil and water), being recognized as a human and animal pathogen worldwide [1–6]. This species has been isolated from a variety of animals, including birds, reptiles, fish, amphibians, invertebrates, wild boar, cattle, seals and armadillos, in addition to domestic animals such as dogs and cats [7]. There are reports from different parts of the world of mycobacteriosis in cats indicating a prevalence of *M. fortuitum* as the etiologic agent. Usually, cats present skin infections in the form of non-healing ulcerative pyogranulomatous dermatitis and panniculitis [7–11]. In other animals, such as cattle, *M. fortuitum* can cause chronic mastitis [12]. In addition, infections caused by *M. fortuitum* are characterized by high resistance to antibiotics, such as aminoglycosides, beta-lactams, macrolides, rifamycins, and tetracyclines [13–15]. Recently, a genomic study revealed that a set of genes associated with resistance to these antibiotics (a*ph(3’’)-Ic*, *aac(2’)-Ib*, *arr*-1, *bla*F, *erm*39, *rbp*A, *rox*, and *tap*) appears to be ubiquitous in *M. fortuitum* species [16]. Since treatment of mycobacteriosis is long-term, isolation and antibiotic susceptibility assays are recommended to avoid delays in treatment [9–11]. Here we present the first reported case of a companion cat (*Felis silvestris catus*) with mycobacteriosis caused by *M. fortuitum* in Brazil.

## Case report

**Fig. 1.**
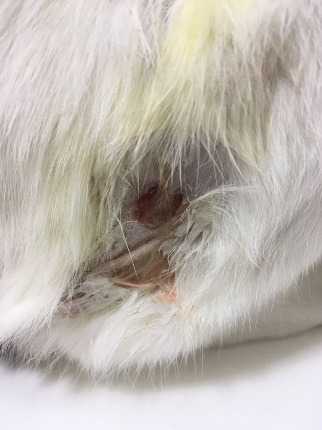
The suppurative and ulcerative aspects of the lesion near the right pelvic limb of the cat.

**Fig. 2.**
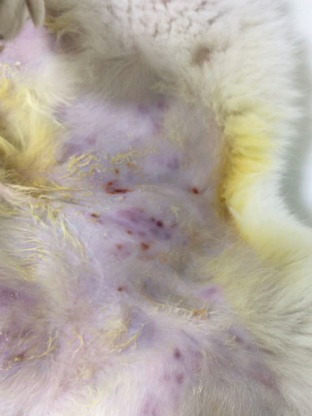
Multiple subcutaneous nodules in the inguinal region of the cat.

An approximately 6-year-old male domestic cat with access to the outdoors (4.8 kg body weight) presented in July 2020 for examination of skin nodules in the lower abdomen, near the right pelvic limb and inguinal region. The cat had been adopted by the owner in 2016, and its exact age and history prior to adoption were unknown. Previously, the cat’s owner had consulted other veterinarians, who performed a biopsy and histopathologic examination suggestive of pyogranulomatous panniculitis. The cat was treated with anti-inflammatory steroids and antimicrobial medications such as amoxicillin with clavulanic acid, sulfamethoxazole with trimethoprim (systemic), and polymyxin B (topical) without success. So, the owner visited a veterinary centre specialising in cats, where new tests were performed. The lesions were firm and painful and had been noticed by the owner about ten months earlier. The masses infiltrated the soft tissue of the right lower abdomen and inguinal region, where they exhibited fistula-like tracts (Figs. 1 and 2). On physical examination, the cat was in good general condition and no other abnormalities were noted. Haematology, blood biochemistry, and electrolytes showed lymphopenia (1340 cells µl^−1^; reference interval 1500–7000 cells µl^−1^) and an increase in serum globulin concentration (6 g dl^−1^; reference interval 2.5–5.1 g dl^−1^). Tests for feline leukaemia virus (FeLV) and feline immunodeficiency virus (FIV) were negative, according to the owner. Tissue samples were collected with a 5 mm punch and sent for repeat histopathologic analysis and bacterial isolation. Histopathologic examination revealed a lesion pattern involving a large portion of the dermis and extending to the adipose panniculus and subcutaneous muscle tissue with a severe inflammatory reaction. This inflammation was characterised by proliferation of mature fibrous connective tissue, oedema, and poorly demarcated nodular inflammatory infiltrates in a coalescing multifocal pattern composed mainly of neutrophils. The findings were suggestive of atypical cutaneous mycobacteriosis. Due to the failure of previous treatment with anti-inflammatories and some antimicrobial drugs, in addition to the suspicion of mycobacteriosis, we performed bacterial isolation and antibiotic susceptibility testing. Bacterial isolation was performed in trypticase soy agar (TSA) culture medium supplemented with 0.05% Tween-80 (Sigma-Aldrich) at 22 °C for 72 h. Taxonomic identification was based on the Multilocus Sequence Analysis scheme with amplification and sequencing of 16S rRNA, *hsp*65 and *rpo*B genes [17], indicating infection by *Mycolicibacterium fortuitum* (named 7G strain). *In vitro* antibiotic susceptibility assays of the *M. fortuitum* 7G strain showed a high rate of resistance to various drug classes based on E-test, with the following MICs: Azithromycin ≥256 µg ml^−1^ and Clarithromycin ≥32 µg ml^−1^ (macrolides); Streptomycin ≥32 µg ml^−1^ and Tobramycin ≥32 µg ml^−1^ (aminoglycosides); Meropenem ≥32 µg ml^−1^ (carbapenem); Cefalotin ≥256 µg ml^−1^ and Cefepime ≥256 µg ml^−1^ (cephalosporins); and Rifampicin ≥32 µg ml^−1^ (rifamycin). Other drugs were also verified by disc diffusion assays (Table 1). Despite the extended antibiotic resistance profile, this strain showed sensitivity to amikacin (aminoglycoside) and ciprofloxacin (quinolone). Therefore, the cat was treated long-term with pradofloxacin (5 mg/kg), an alternative quinolone, because the bioavailability of ciprofloxacin in cats is low.

**Table 1.** Antibiotic susceptibility profile based on disk diffusion

Antibiotic class	Antibiotic	Resistance
Aminoglycosides	Amikacin, Gentamicin, Kanamycin, Neomycin	S
	Streptomycin, Tobramycin	R
Carbapenems	Ertapenem, Imipenem, Meropenem	R
Cephalosporins 1Gen	Cephalotin	R
Cephalosporins 2Gen	Cefoxitin	S
Cephalosporins 3Gen	Cefotaxime, Ceftriaxone	R
Cephalosporins 4Gen	Cefpirome	R
Macrolides	Azithromycin, Clarithromycin, Spectinomycin	R
Penicillins	Amoxicillin, Ampicillin-Sulbactam, Carbenicillin, Oxacillin, Penicillin G, Piperacillin-tazobactam	R
Quinolones	Nalidixic Acid	R
	Ciprofloxacin, Ofloxacin, Levofloxacin, Norfloxacin	S
Sulfonamides	Sulfametaxazole, Sulfametaxazole-Trimetroprime	S
	Trimetroprime	R
Tetracyclines	Minocycline, Tetracycline	R
Monobactams	Aztreonam	R
Glycopeptide	Vancomycin	R
Lincosamide	Clindamycin, Lincomycin	R
Others	Chloramphenicol, Fosfomycin, Nitrofurantoin, Rifampicin, Ticarcillin-clavulanic	R
	Tigecycline	S

S, susceptible; R, resistant.

To gain additional insight into the presence of other antibiotic resistance determinants in this *M. fortuitum* 7G strain, we performed sequencing of its genome. Genomic DNA was extracted (100 ng) using NucleoSpin Microbial DNA (Macherey-Nagel) and sequenced using the Nextera XT library preparation kit on the Illumina HiSeq 2500 platform with 2×250 bp paired-end reads. Genomic analysis using Comprehensive Antibiotic Resistance Database v.3.1.3 [18] revealed the presence of several antibiotic resistance genes, such as *aph*(3'')-Ic (aminoglycoside resistance), *arr*-1 (rifamycin), *bla*F (β-lactamase), *erm*39 (macrolide), *aac*(2')-Ib (aminoglycoside), and *rbp*A (rifamycin). In addition, we identified four secretion systems of the VII type (ESX-1, ESX-3, ESX-4, and ESX-4-bis) that have previously been associated with virulence and survival in *Mycobacterium* [19].

## Discussion

Cases of mycobacterial disease associated with non-tuberculous mycobacteria are increasing worldwide in humans and animals [1, 5]. Companion animals, especially cats and dogs, that are susceptible to these infections may pose a public health risk. In addition, mycobacteriosis can be difficult to diagnose and lengthy to treat [9, 11, 20]. A common aetiological agent in mycobacterial disease is *Mycolicibacterium fortuitum *, an organism ubiquitous in the environment with a typical clinical presentation in skin and soft tissue [21]. Therefore, the identification of a multidrug-resistant strain of *M. fortuitum* causing disease in a companion cat in Brazil demonstrates the importance of proper diagnosis and treatment of animal diseases for the one health. Although histopathological examination is a reliable method for diagnosing cutaneous mycobacteriosis, without culture and molecular analysis, the aetiological agent of the infection could not have been diagnosed as *M. fortuitum *. Furthermore, proper antibiotic treatment is crucial in this matter as it has an impact on the prompt recovery of the animal and the presence of this pathogen in this environment. Here, the antibiotic susceptibility profile of the 7G strain was determined *in vitro* using disc diffusion and E-test to provide appropriate antibiotic therapy. Interestingly, this strain showed resistance to rifampicin, which is worrying as this drug is used worldwide as first-line therapy for human tuberculosis [22]. In addition, our study provided the first genome of an *M. fortuitum* strain associated with animal disease. Through genomic analysis, we identified several genes on the chromosome associated with antibiotic resistance, suggesting that they are inherent to this organism and not related to plasmid-mediated acquisition. Indeed, in a previous *in silico* study of *Mycolicibacterium* plasmids, no notable antibiotic resistance genes were observed in plasmids of *M. fortuitum* [23]. Another aspect verified in the genome of the 7G strain was the type VII, present in four loci and reported to be associated with virulence, iron uptake and conjugation in several species of the *Mycobacteriaceae* family [24]. Thus, these systems could favour the adaptation and colonisation of the *M. fortuitum* strain in different environments.

In conclusion, culture and molecular analysis of mycobacterial infections accelerate diagnosis and allow appropriate treatment and control of the aetiological agent.
